# Psoriasis and neurodegenerative diseases—a review

**DOI:** 10.3389/fnmol.2022.917751

**Published:** 2022-09-26

**Authors:** Julia Nowowiejska, Anna Baran, Iwona Flisiak

**Affiliations:** Department of Dermatology and Venereology, Medical University of Bialystok, Bialystok, Poland

**Keywords:** psoriasis, neurodegenerative disorders, neurodegenerative diseases, Alzheimer's disease, Parkinson's disease, amyotrophic lateral sclerosis, oxidative stress

## Abstract

Psoriasis is a chronic skin disease with underlying genetic, inflammatory and immunological background, which is a great medical problem, currently regarded as a systemic condition. Neurodegenerative diseases (NDs) are characterized by a progressive loss of nervous tissue, which affects elderly people more frequently; therefore, it is suspected that, due to society's aging, morbidity is going to increase. We performed a thorough review in order to investigate for the first time whether psoriasis may predispose to different particular neurodegenerative diseases—Alzheimer's disease (AD), Parkinson's disease (PD), and amyotrophic lateral sclerosis (ALS). PubMed search resulted in the retrieval of 833 records, of which 77 eligible were included in the review. Our thorough analysis revealed there are some potential links between psoriasis and NDs (inflammation, oxidative stress, genetics, cardiometabolic disorders), but there is no strong evidence that psoriasis may predispose to NDs. Based on the evidence, it seems that the risk of PD in psoriatics is not increased, and the evidence for increased risk of AD slightly prevails the data that state the opposite. ALS risk does not seem to be increased in psoriatics. The paucity of original studies does not allow for the formulation of definitive conclusions but encourages to perform further investigations.

## Introduction

Psoriasis is a chronic skin disease that affects about 125 million people worldwide (NPF., 2021). It is an essential medical problem since nowadays it is perceived as a systemic disease due to its wide comorbidity (Nowowiejska et al., 2021). Moreover, psoriasis is also a crucial social issue because it decreases quality of life of patients significantly and leads to physical impairment and inability to work (Bulat et al., 2020). The pathogenesis of psoriasis is multifactorial. Genetic, as well as immunological and environmental factors are considered (Baran et al., 2021). There are many chromosomal loci identified, which provide susceptibility for psoriasis development (Rendon and Schäkel, 2019). Moreover, the essential feature of psoriasis is sustained inflammatory condition, which leads to uncontrolled keratinocyte proliferation and abnormal differentiation, as well as neovascularization (Rendon and Schäkel, 2019). The immune pathways in psoriasis involve antimicrobial peptides (AMPs), dendritic cells (DCs), Th17 lymphocytes, tumor necrosis factor (TNF) α, interleukins (IL) 17, 22, 23, and signal transducer and activator of transcription (STAT) 3 (Tokuyama and Mabuchi, 2020). Flares of psoriasis may be triggered by several environmental stimuli, such as stress, infection, alcohol consumption, or particular medicine intake (Baran et al., 2021). As for the treatment, there are numerous methods, including topical as well as systemic options (Nowowiejska et al., 2020). Systemic treatment includes either classic antipsoriatic agents (eg. methotrexate, cyclosporin A, or acitretin) or biologics—monoclonal antibodies or receptor fusion proteins (Rendon and Schäkel, 2019). Biologic agents usually target TNF-α, IL-12, 17, 23, or their receptors (Rendon and Schäkel, 2019).

Neurodegenerative diseases (NDs) are defined as disorders that are characterized by the progressive loss of nervous tissue (Agrawal and Biswas, 2015). There is an association between increased morbidity and advanced age; therefore, it is suspected that the frequency of NDs occurrence is going to increase nowadays due to societal aging (Agrawal and Biswas, 2015). The most common and well-known diseases considered among this group are Alzheimer's disease (AD), Parkinson's disease (PD), and amyotrophic lateral sclerosis (ALS) (Agrawal and Biswas, 2015). Their pathogenesis is different and various parts of the nervous system may be affected by the pathological process but all of them result in the loss of neuronal tissue (Agrawal and Biswas, 2015).

Alzheimer's disease is the most frequent cause of dementia, responsible for ~50–75% of all its cases (Lane et al., 2018). Most AD cases are sporadic but there are several known risk factors: advanced age, Down's syndrome, genetic predispositions, or apolipoprotein E gene polymorphism (Brown et al., 2005). The hallmark of AD is amyloid plaques and neurofibrillary tangles (Lane et al., 2018). The accumulation and oligomerization of amyloid influence synaptic efficacy, which leads to microglial and astrocytic activation and neuroinflammation, followed by oxidative injury and disturbed ionic homeostasis, all resulting in widespread neuronal dysfunction and loss of nervous tissue (Lane et al., 2018). AD manifests clinically mostly with progressive problems with episodic memory, followed by difficulties in multitasking and mobility and behavioral alterations (Lane et al., 2018). Unfortunately, there is no disease-modifying therapy. Treatment focuses on the use of symptomatic drugs, such as acetylcholinesterase inhibitors, memantine or antidepressants, and neuroleptics, if needed (Lane et al., 2018).

Parkinson's disease is probably the second most common ND, which affects ~4 million people worldwide (Hayes, 2019). It occurs more frequently in men than women and identified risk factors are advanced age, family history, living in a rural environment, and exposure to pesticides (Beitz, 2014; Hayes, 2019). The primary cause of PD is not known but the essence of the disease is the depigmentation of substantia nigra and locus coeruleus and dopaminergic neuron loss which occur through the apoptosis and autophagy processes (Beitz, 2014; Hayes, 2019). The hallmark of PD in affected areas of the brain is the presence of Lewy bodies containing α-synuclein (Hayes, 2019). PD is mostly characterized by motor symptoms, of which the most common are: resting tremor, bradykinesia, postural instability, and rigidity (Hayes, 2019). Moreover, PD may present with other, non-motor symptoms, such as cognitive impairment, autonomic dysfunction, depression, or anxiety (Beitz, 2014; Hayes, 2019). The treatment usually consists of dopaminergic drugs, of which the first was levodopa (Hayes, 2019).

Amyotrophic lateral sclerosis, also called Lou-Gehrig's disease, is another ND that is characterized by the progressive deterioration and loss of function of the motor neurons in the central nervous system (Hulisz, 2018). ALS can be either familial or sporadic (idiopathic) (Hulisz, 2018). Similar to previous NDs, the pathogenesis of ALS is complex and still not fully understood but aberrant RNA metabolism, impaired nucleocytoplasmic and endosomal transport, abnormal axon structure and function, excitotoxicity, neuroinflammation, and mitochondrial dysfunction are taken into account (Brown and Al-Chalabi, 2017). Independently on the mechanism, the endpoint in ALS is the dysfunction of motor neurons, which lead to the denervation of target cells (Brown and Al-Chalabi, 2017). The first clinical symptom of ALS is usually progressive, unilateral weakness and spasticity in the distal parts of lower and upper limbs or fasciculations, cramps, and muscle wasting (Brown and Al-Chalabi, 2017; Hulisz, 2018). As for cognitive impairment, it is estimated that up to 50% of patients maintain their cognitive functions throughout the course of ALS (Brown and Al-Chalabi, 2017). Currently, there are two modifying therapies for ALS, and symptomatic treatment is used (Brown and Al-Chalabi, 2017).

While analyzing the pathogenesis of psoriasis, we encounter many links between this skin disease and some of the most common NDs. Noteworthy is the fact that skin and nervous tissue come from the same germ layer—ectoderm—and many diseases may present with symptoms involving skin and nervous system at the same time.

To the best of our knowledge, this is the first review of the possible associations between different NDs and psoriasis. The review aimed at collecting all possible indicators of the associations between psoriasis and NDs in order to draw conclusions about their potential relationship that could be useful in screening psoriatics and adjusting their treatment.

## Search strategy

**Figure 1:**
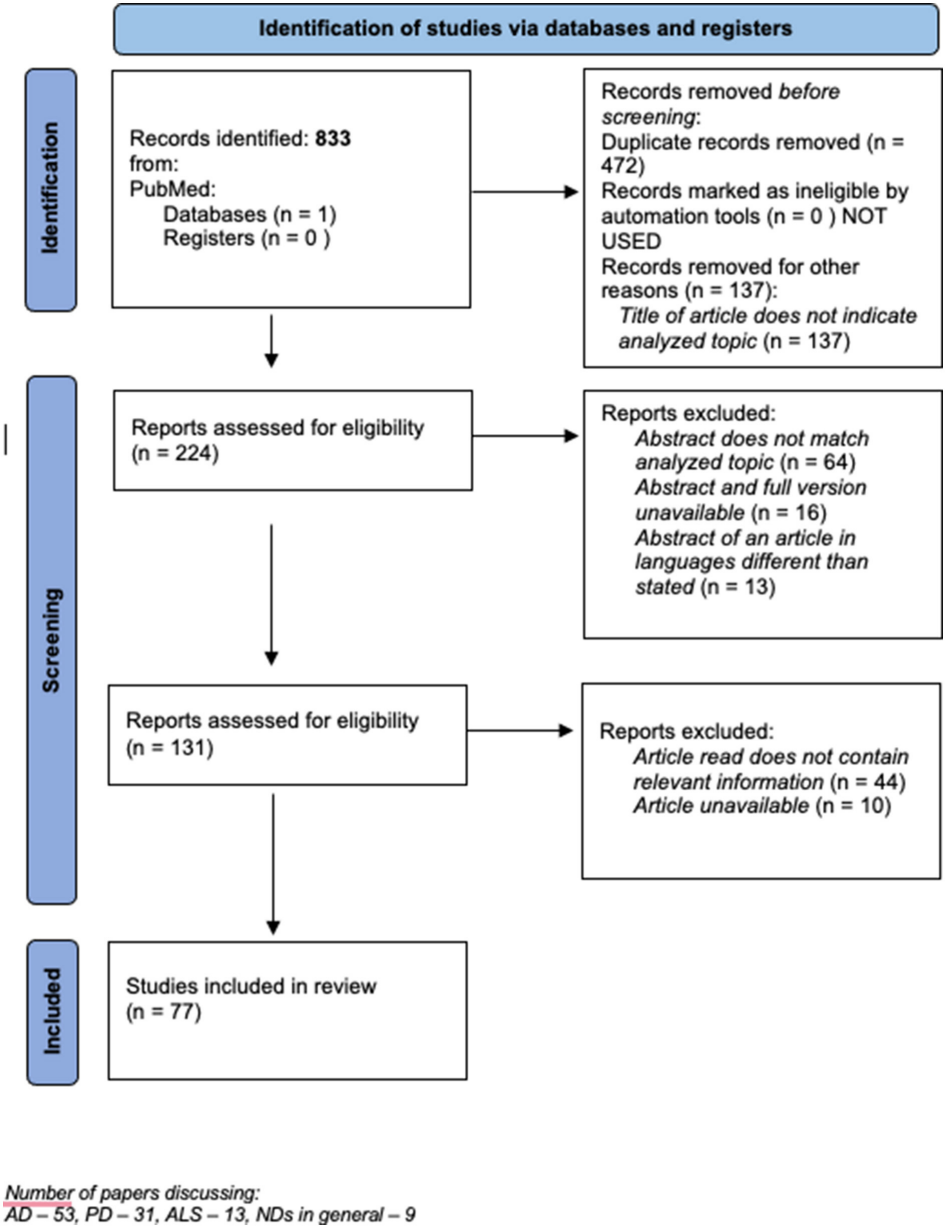
The method of the search strategy. Number of papers discussing : AD = 53, PD = 31, ALS = 13, NDs in general = 9.

We performed the review according to the following scheme (PRISMA Guidelines., 2021). The literature search through PubMed, conducted on November 19–27, 2021, was performed using relevant medical subject headings (MeSH) with all subheadings included and without date limitations. The main subject of the research was psoriasis and neurodegenerative diseases. We took into consideration the most frequent neurodegenerative disorders: Alzheimer's disease, Parkinson's disease, and amyotrophic lateral sclerosis. MeSH terms included: “psoriasis” and “neurodegenerative” or “neurodegenerative disorders” or “neurodegenerative diseases” or “Alzheimer” or “Alzheimer's disease” or “Parkinson's” or “Parkinson's disease” or “amyotrophic lateral sclerosis” or “ALS” or “Lou-Gehrig's disease.” We read the abstract of each article whose title suggested the association between psoriasis and neurodegenerative disorders. The whole article was read when the abstract indicated that the article covers our topic. Articles written in the following languages were taken into consideration: English, German, French, Polish, and Hungarian. Other languages were excluded. The database was searched by two authors (J. N. and A. B.) and then checked by the third author (I. F.). The results of every search were combined, and duplicates were removed. Finally, all selected eligible articles were fully reviewed. Due to the paucity of data, we included original articles, review articles, case reports including a brief review of the topic and letters to editor, as well as human and animal studies. Moreover, due to the very small number of research concerning specifically the association between psoriasis and neurodegenerative diseases, we included all articles which contained any information regarding this possible relationship that could explain their common background. The method of the search strategy is presented in Figure 1.

## Retrieved articles

The search resulted in the retrieval of 833 records of which 224 were included for abstract analysis, then 64 were removed because the abstract did not match the topic, 16 because the abstract and full version of the article were not available and 13 because the abstract indicated that the language of the full version of the article is other than assumed. Then 131 articles were selected to be read in the full version, 10 were removed due to the lack of access to the full version, and 44 because the article did not contain relevant information. 77 articles were ultimately included in qualitative synthesis, of which 49 are review articles, 22 are original articles, 2 case reports, and 4 letters to editor. The whole list of articles taken into consideration and their key outcomes are presented in Supplementary Table 1.

## Discussion

There is a great paucity of data focusing on the association, particularly between psoriasis and NDs. There are only 22 original research discussing psoriasis and NDs at the same time, and even less investigating this matter on purpose. Other articles usually discuss different pathogenic paths presenting diseases in which they may be involved. Therefore, not only did we focus on specific original findings but we also analyzed different, potentially common elements in the pathogenesis of these two entities, as well as some medicines which are successful in the treatment of both of them, which could indicate similar background. Based on our thorough analysis, there are some potential links between psoriasis and NDs, but there is no strong evidence that psoriasis may predispose to NDs. We would like to address a few most important issues below.

### Existing original studies directly regarding the association between psoriasis and neurodegenerative disorders

We identified only 11 original studies directly investigating the association between psoriasis and particular NDs: 6 of them concerning AD, 4 concerning PD, and 1 concerning ALS, AD, and PD. Their main strength is that all of them were human studies and the majority were based on large sample size.

#### Alzheimer's disease and psoriasis

Kim et al. investigated the risk of AD in 535,927 patients with psoriasis. In a multivariable-adjusted model, psoriatics had a significantly increased risk of AD (HR 1.09; 95% CI, 1.07–1.12) compared to the control group without psoriasis. The influence of systemic antipsoriatic treatment was also studied. It has been shown that psoriatic patients who did not receive systemic agents had significantly increased risk of AD compared to patients who were treated systemically (HR 1.10; 95% CI, 1.08–1.12 vs. HR 0.99; 95% CI: 0.90–1.09) (Kim et al., 2020), which may indicate a role of antipsoriatic therapy in AD course and is going to be discussed below. Another large study, a retrospective analysis, was conducted on 56 million adults, including 309,660 subjects with psoriasis, to find out whether anti-TNFα agents affect the risk of AD development (Zhou et al., 2020). Psoriasis was associated with a higher risk of AD and dementia (AOR = 1.37 (1.31–1.42), *p* < 0.0001) (Zhou et al., 2020). Anti-TNFα agents were associated with decreased risk for comorbid AD in patients diagnosed with psoriasis (Zhou et al., 2020). Lai et al. aimed to investigate whether autoimmune comorbidities, including psoriasis, predispose patients with Down's syndrome to earlier onset of AD, as this genetic syndrome is known to be associated both with AD and autoimmune diseases (Lai et al., 2021). Based on the group of 339 patients with Down's syndrome, of which 34 had also psoriasis, they did not find a significant influence of psoriasis on the time of AD onset (Lai et al., 2021). The main disadvantage would be the small sample size (*n* = 34). Another research aimed to compare cognitive impairment and risk of dementia between 318 psoriatics and 9,678 non-psoriatics and stated that psoriasis was not associated with preclinical markers or higher dementia risk (Pezzolo et al., 2021). The study by Wotton et al. intended to obtain information on whether admission to a hospital with an autoimmune disease (including 3,039 subjects with psoriasis) is associated with an increased risk of dementia (Wotton and Goldacre, 2017). The risk of AD was elevated in patients admitted to the hospital for psoriasis (1.08, 1.01, to 1.17, *p* < 0.03) (Wotton and Goldacre, 2017). Data obtained from genome-wide association studies (GWAS) seem to bring inconsistent information. First study aimed to systematically investigate the genetic overlap between AD and psoriasis using summary data from GWAS at multiple academic clinical research centers (Yokoyama et al., 2016). They found single nucleotide polymorphisms (false discovery rate *p* < 0.05) were associated with both AD and immune-mediated diseases, of which rs2516049 (closest gene HLA-DRB5; conjunction false discovery rate *p* = 0.04 for AD and psoriasis, 5.37 × 10^−5^ for AD, and 6.03 × 10^−15^ for psoriasis) demonstrated the same direction of allelic effect between AD and psoriasis (Yokoyama et al., 2016). In the second GWAS, no shared genetic loci were found between psoriasis and AD (Li et al., 2021).

#### Parkinson's disease and psoriasis

Witoelar et al. conducted a study on 138,511 individuals of European ancestry where they looked for common genetic risk variants conveying the risk of PD and autoimmune diseases and to identify new shared genetic variants (Witoelar et al., 2017). One of these autoimmune disorders was psoriasis. The authors reported the decreased risk for psoriasis but they pointed out that their findings were not statistically significant due to the small sample size (Witoelar et al., 2017). One study retrospectively compared the prevalence of PD in two groups of ethnic Poles, with bullous pemphigoid and with psoriasis (Bartkiewicz et al., 2017). They enrolled 96 patients with pemphigoid and 149 with psoriasis. They observed a higher prevalence of PD only in patients with pemphigoid (Bartkiewicz et al., 2017), but the limitation of the study was the small sample size and only one nationality taken into account. Lee et al. performed a nationwide population-based cohort study on 548,327 patients with psoriasis to investigate the frequency and risk factors of PD in psoriatics (Lee et al., 2020). The patients with psoriasis presented a significantly increased risk of PD development (HR 1.091, 95% CI 1.029–1.115). Interestingly, the risk of PD was significantly elevated in psoriatic patients who were not treated with systemic therapy (HR 1.093, 95% CI 1.031–1.159) and decreased among the psoriatics who received such therapy (HR 1.04, 95% CI 0.806–1.316) (Lee et al., 2020), which may again point into some role of antipsoriatic treatment. Li et al. found no shared genetic loci between psoriasis and PD in GWAS (Li et al., 2021), similar to AD. One study assessed the risk of parkinsonism during a 5-year follow-up after the diagnosis of psoriasis in 4,885 patients and concluded that they were at significantly increased risk (Sheu et al., 2013).

#### Amyotrophic lateral sclerosis and psoriasis

We identified only one original study focusing specifically on the association of ALS with psoriasis (among other autoimmune diseases)—research conducted by Li et al. which was mentioned above. The authors found no shared genetic loci between psoriasis and ALS (Li et al., 2021).

### Existing review articles regarding the association between psoriasis and each neurodegenerative disorder separately or neurodegenerative disorders in general

We managed to find 4 review articles investigating the association of psoriasis with different particular NDs separately. The first two articles concerned AD and were rather in favor of the association between these two entities. An article, by Zhao et al., reviewed the possibility that psoriasis increases the risk of dementia (Zhao et al., 2021). They concluded that most studies support such a hypothesis, but of course, more studies are required for definite conclusions (Zhao et al., 2021). A review by Zhang et al. introduced a hypothesis of a bidirectional link between psoriasis and AD: on one hand, psoriasis predisposes to AD, but on the other hand, AD patients may be at increased risk of psoriasis (Zhang et al., 2021). The authors justified the relationship between these two diseases by the common background: inflammatory, immunological, and genetic (Zhang et al., 2021). In the last two reviews, which concerned PD, there were discrepancies. Ungprasert et al. (2016) and Amanat et al. (2018) reviewed the potential links between psoriasis and PD. The first author stated that there are inconsistent data, whereas the second author suggested a significantly increased risk of PD among psoriatics (Ungprasert et al., 2016; Amanat et al., 2018).

Due to the paucity of data from original studies, we decided to take into consideration all other research, which might shed some light on the possible relationship between psoriasis and NDs. Therefore, we investigated the below-mentioned aspects of this association.

### Genetics

Genetic factors have been taken into consideration in the pathogenesis of psoriasis and some of the NDs for a long time. Indeed, genetic predisposition has been proved for psoriasis and AD, PD, and ALS (Brown et al., 2005; Hulisz, 2018; Baran et al., 2021). Besides the original studies on genetic issues in psoriasis described above, we have also found some evidence considering the common genetic backgrounds for psoriasis and NDs. Probably, the most famous association between these two entities is the apolipoprotein E. Its function is lipid transport but it is also able to affect immune cells and cytokine secretion (Zhang et al., 2011). It has not only an indisputable role in AD pathogenesis but also it is involved in PD and psoriasis (Zhang et al., 2011). Mutations in Zdhhc family genes lead to changes in palmitoylation or de-palmitoylation, which may result in NDs and inflammatory diseases, including psoriasis (Zhou et al., 2021). One study aimed to characterize the co-expression clustering in mammalian genomes. They chose to investigate AD and psoriasis and concluded that chromosome territory reorganization may play a role in the course of both (Woo et al., 2010). D'Amico et al. investigated whether there is an association between rs2294020 in X-linked CCDC22 and susceptibility to different diseases, including psoriasis and ALS (D'Amico et al., 2017). They found a significant association between the occurrence of this SNP and the susceptibility to psoriasis and ALS (D'Amico et al., 2017). Theotoki et al. investigated the role of dicer—RNA-binding protein (RBP) that can form complexes and influence the transcription process (Theotoki et al., 2020). Its role has been demonstrated in many diseases including psoriasis and PD, although in these two it acts opposite. In psoriasis, the dicer is upregulated and the abnormal expression of this protein may be associated with the dermatosis progression. In PD, downregulation of dicer occurs and leads to increased cell death, exacerbation of inflammation, and appearance of PD clinical symptoms (Theotoki et al., 2020).

### Oxidative stress

Oxidative stress occurs due to the imbalance between the production of reactive oxygen species (ROS) and their elimination (Srivastava et al., 2017). This process is unfavorable since it contributes to the damage of cells and tissues. In the natural setting, ROS are the side products created in the human organism, mainly in mitochondria, due to oxygen metabolism (Bhattacharyya et al., 2014). Moreover, different factors may stimulate their formation, these are for instance: ultraviolet radiation, ionizing radiation, alcohol, cigarettes, metals, and drugs (Bhattacharyya et al., 2014). When the concentration of ROS remains within normal limits, they may be even considered beneficial particles, since they play role in the immune processes and signal transmission (Bhattacharyya et al., 2014). The problem occurs when the production of ROS is too intensive. Under such circumstances, structural alterations in basic organic compounds occur in proteins, lipids, and nucleic acids. The role of oxidative stress has been proved in many different diseases, including neoplasms, diabetes mellitus, atherosclerosis, cardiovascular diseases, kidney diseases, respiratory system, and NDs (Bhattacharyya et al., 2014). The maintenance of redox homeostasis in the nervous system is crucial for the prevention of NDs (Rosito et al., 2020). Endogenous substances acting against oxidative stress, the so-called antioxidants, are glutathione, Q10 coenzyme, L-arginine, as well as enzymes: superoxide dismutase (SOD), glutathione peroxidase, and catalase. Among exogenic substances of similar potency, we consider vitamin E and flavonoids (Pizzino et al., 2017).

Oxidative stress has been proved to be an important element in psoriasis pathogenesis (Srivastava et al., 2017). Similarly, it has to be mentioned in some NDs. In psoriasis, oxidative and nitrosative stress pathways and elevated ROS are associated with psoriatic skin inflammation (Maes et al., 2011). Decreased concentrations of SOD, catalase, and PON1 have been noted (Srivastava et al., 2017). Noteworthy, some studies suggested a positive correlation with psoriatic skin lesion severity with markers of oxidative stress and a negative correlation with antioxidants (Lin and Huang, 2016). In NDs, the role of oxidative stress is prominent since nervous tissue is characterized by great oxygen consumption (Chiurchiù and Maccarone, 2011). Microglial activation, which occurs in both—AD and PD—leads to the secretion of pro-inflammatory cytokines and ROS, neuroinflammation, and neurodegeneration (Maes et al., 2011; Srivastava et al., 2017). The accumulation of amyloid protein in AD leads to ROS generation, lipid and DNA peroxidation, microglia and astrocytes activation, the release of inflammatory cytokines, cell death, and loss of nervous tissue, which is the essence of neurodegeneration (Chiurchiù and Maccarone, 2011). In PD, oxidative stress induced by dopamine contributes to the accumulation of α-synuclein, direct damage of neurons, and dysfunction of mitochondria (Chiurchiù and Maccarone, 2011). In patients with ALS, especially those of familial origin, the mutation of the *SOD* gene may have many effects, and one of them is the enhancement of oxidative stress followed by the misfolding of the mutant SOD product and the creation of abnormal protein aggregates (Chiurchiù and Maccarone, 2011). Research has shown that two other important pathogenic elements in ALS, namely excitotoxicity and defective axonal transport, may be also the consequence of oxidative stress (Chiurchiù and Maccarone, 2011). Studies have shown, that in both, psoriasis and NDs, SOD expression is altered (Gerbaud et al., 2005). Manganese SOD in psoriatics and NDs scavenges generated free radicals (Li and Zhou, 2011), and there is increased activity in manganese and copper/zinc SOD in human dermal psoriatic fibroblasts (Gerbaud et al., 2005).

4-hydroxy-2-nonenal (4-HNE) is called ‘the second messenger of reactive oxygen species' and is suspected as a biomarker of oxidative stress (Jaganjac et al., 2020). It is a growth-regulating factor involved in redox signaling. 4-HNE can modify proteins and alter signaling pathways involved in normal cellular functioning, and therefore, contribute to the pathogenesis of NDs. The role of 4-HNE was described in AD, PD, and ALS (Jaganjac et al., 2020). It was also investigated in psoriasis and it appears that 4-HNE has the properties of a signaling molecule, which can affect key elements of psoriasis pathogenesis: proliferation, differentiation, and apoptosis (Jaganjac et al., 2020).

Vascular adhesion protein 1 (VAP-1) is a member of the amine oxidases enzymes family, takes part in leukocyte extravasations, and has an insulin-like influence on the metabolism of glucose through hydrogen peroxide, which excess contributes to oxidative stress (Nurminen et al., 2011). VAP-1 is involved in the pathogenesis of many diseases, including psoriasis and AD, and its inhibitors may be used as a therapeutic target (Nurminen et al., 2011).

Two articles by Das investigated the role of angiotensin II (Das, 2005a,b), as it stimulates the release of ROS, followed by the activation of NF-κB (nuclear factor kappa-light-chain-enhancer of activated B cells) and secretion of pro-inflammatory cytokines. It was suggested that in conditions with low plasma polyunsaturated fatty acids (PUFAs) concentrations, namely AD and psoriasis, increased activity of the angiotensin-converting enzyme (ACE) could occur, which leads to the transformation of angiotensin I into angiotensin II, which results in the inflammatory state (Das, 2005a,b).

**Figure 2:**
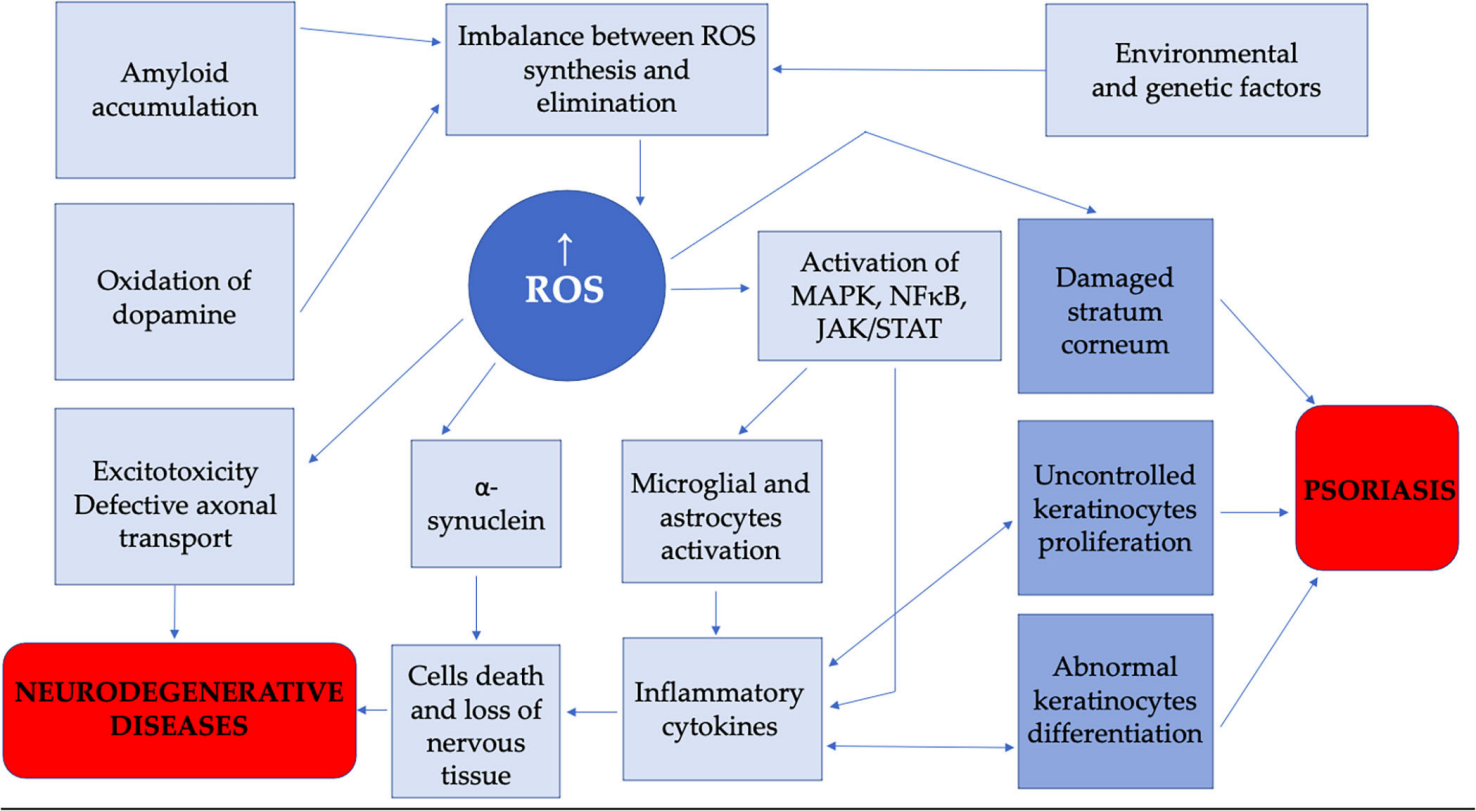
The role of oxidative stress in pathogenesis of psoriasis and neurodegenerative diseases.

The role of oxidative stress in the pathogenesis of psoriasis and NDs is presented in Figure 2.

### Inflammation

Inflammation has an undeniable role in the pathogenesis of psoriasis, which is currently perceived not only as a skin disease but even as a chronic, general, inflammatory condition (Schultze and SYSCID consortium, 2018; Baran et al., 2021). Considering the tight association between psoriasis and metabolic syndrome (MS), there has been a term called “metaflammation” proposed, which indicates metabolically induced inflammation in psoriatic patients (Nowowiejska et al., 2020). Pro-inflammatory cytokines, whose expression is upregulated in psoriasis, lead to hyperproliferation and abnormal differentiation of epidermal cells, which are the hallmark of this dermatosis. Moreover, they promote the synthesis of chemokines and AMP, which consecutively further exacerbate the release of inflammatory cytokines in a vicious circle manner. These molecules influence immune cells leading to sustained skin inflammation (Lin and Huang, 2016). Chronic inflammation has also been demonstrated in NDs (Schultze and SYSCID consortium, 2018).

The involvement of particular cytokines in the pathogenesis of psoriasis and NDs is relevant (Bougea et al., 2014; Chen et al., 2020), of which IL-17 seems the most prominent. IL-17 is a whole family of particular cytokines labeled A-F. They are engaged in immune response, especially against extracellular fungi, and also play an important role in the inflammatory condition in several allergic or autoimmune diseases, such as psoriasis, lupus erythematosus, or rheumatoid arthritis (Monin and Gaffen, 2018). The key role of IL-17A in psoriasis has been proved by the upregulation of *IL-17A* and related genes in affected and non-affected skin of psoriatics and secretion of IL-17A by cells involved in psoriasis pathogenesis (Blauvelt and Chiricozzi, 2018). One of the sources of IL-17A in psoriasis is Th17 cells, then they are stimulated to secrete further cytokines by IL-23, which include (besides IL-17A): IL-17F, IL-21, IL-22, IL-26, and TNF-α. Of note, an elevated number of Th17 lymphocytes is observed in the blood and lesional skin of patients with psoriasis. Other sources of IL-17A may be mastocytes, γδ T cells, αβ T cells, and innate lymphoid cells (Blauvelt and Chiricozzi, 2018). In the review article by Chen et al., information on the important role of IL-17 in psoriasis was provided, as well as in AD, PD, and ALS (Chen et al., 2020). Moreover, Bougea et al. in their case report of the patient highlighted the role of IL-17 in psoriatic arthritis and in the pathology of the spinal cord in ALS patients (Bougea et al., 2014). Indeed, the role of IL-17 has been studied also in NDs. It was reported that Th17 cells exacerbate neuroinflammation and neurodegeneration in a rodent model of PD. An increased proportion of circulating Th17 lymphocytes was also observed in patients with mild cognitive impairment, and in persons with AD elevated levels of IL-17 in serum were associated with disease progression and Th17 lymphocytes were found to infiltrate the brain of AD models, stimulating inflammation and death of neuronal cells. Interestingly, IL-17 is thought to trigger AD onset even independently of Aβ or tau protein pathology (Brigas et al., 2021).

Another important cytokine discussed in relation to psoriasis and neurodegeneration is IL-10, which was investigated in the curcumin model (described more thoroughly below) (Mollazadeh et al., 2019). There is also IL-22 role mentioned by Xin et al. (2015). In their review, they described a study (Saresella et al., 2011) in which immunophenotypic and functional analysis of amyloid-stimulated T cells in patients suffering from AD was performed. They found out that IL-5, 21, and 23 are engaged in the differentiation of Th17 lymphocytes with the associated secretion of IL-21 and 22, and postulated that IL-22 may be an important cytokine involved in neuroinflammation in AD (Xin et al., 2015). As described in the introduction, IL-22 takes part in the pathogenesis of psoriasis as well and apparently, it was the first disease associated with dysregulated secretion of this cytokine. Th17 cells, which are known to play an essential role in immunological disturbances in psoriasis, secrete IL-22. Increased levels of this interleukin are found in psoriatic skin lesions and their serum. The result of IL-22 activity is the promotion of cell proliferation, inhibition of their differentiation, and chemotaxis of inflammatory cells (Xin et al., 2015). Another key cytokine, which is engaged in the pathogenesis of psoriasis and also NDs, is mentioned earlier TNFα (Yu et al., 2008). Its inhibitors are approved for psoriasis treatment, and there are also reports on their beneficial role in NDs (described below).

As for particular cell involvement, Th17 lymphocytes and pro-inflammatory CD4+ T cell lineage, are an important link in psoriasis, as described in the introduction, but apparently also in PD (Storelli et al., 2019). One systematic review highlighted the fact that psoriatics may have a 38% increased risk of PD, probably due to the chronic inflammatory condition (Storelli et al., 2019). Another type of cell that participates in psoriasis and NDs pathogenesis are macrophages. Macrophages play an important role in psoriasis pathogenesis since they are activated by pro-inflammatory cytokines and release TNFα and IL-23 and exacerbate inflammation even more (Schultze et al., 2015). As discussed above, psoriasis and NDs have similar cytokine and cell profiles engaged in their pathogenesis which may indicate their mutual relationship and suggest that subjects with psoriasis may be actually more likely to develop NDs, especially considering psoriasis has an earlier average time of onset compared to NDs, and hence, long-term disease and pathological immune processes duration perhaps may favor neurodegeneration.

Another common link between psoriasis and NDs is α7 nicotinic acetylcholine receptors. They are expressed on macrophages and immune cells (Bencherif et al., 2011). When acetylcholine binds with these receptors it results in the downregulation of pro-inflammatory cytokines release and alleviation of inflammatory processes. Microglial cells are characterized by the expression of such receptors and administration of acetylcholinesterase, which increases acetylcholine availability, was demonstrated to attenuate neuroinflammation. Research has shown that nicotine has a neuroprotective influence on dopaminergic neurons through an anti-inflammatory mechanism associated with the modulation of microglial activation and studies with nicotine pretreatment decreased this activation. As these effects were inhibited by the α7 nicotinic receptor antagonist, it confirmed the essential role of α7 receptors in PD pathogenesis. They were also suggested as important in psoriasis and psoriatic arthritis as they were highly expressed in the perivascular setting of synovial tissues in the majority of psoriatics. All this evidence indicates that α7 nicotinic acetylcholine receptors could become therapeutic targets in psoriasis or NDs (Bencherif et al., 2011). As another potential therapeutic target could be voltage-gated potassium channel KV1.3. for venom-derived peptides considered (Tajti et al., 2020). Microglia, which are involved in the pathogenesis of AD, express such channels in the central nervous system, which are necessary for microglial proliferation, so the idea appeared to invent agents affecting these channels to regulate neuroinflammatory processes. The attempt to block these channels was also taken in psoriasis (Tajti et al., 2020).

The role of the complement system, particularly C5 receptor, has been implicated in the pathogenesis of psoriasis and AD (Robertson et al., 2018). The activation of the complement pathway due to different stimuli, eg. infection or tissue damage, in general leads to the release of anaphylatoxins—C5a and C3a. C5a has pro-inflammatory properties expressed through the receptor C5a anaphylatoxin chemotactic receptor 1 (C5aR1/CD88) present on myeloid origin cells. Apparently, this cascade is also important in NDs and psoriasis, and inhibitors of this pathway could be used as drugs in these conditions (Robertson et al., 2018).

A particular interest is given to different plants, which have a positive impact on both, psoriasis and NDs, *via* counteraction against the inflammatory processes, which could further indicate their common background. The most frequently mentioned is turmeric (Aggarwal and Shishodia, 2004; Shishodia et al., 2005; Goel et al., 2008; Hatcher et al., 2008; Pari et al., 2008; Shishodia, 2013; Mollazadeh et al., 2019; Akaberi et al., 2021). It is obtained from the root of *Curcuma longa* and its active ingredient is curcumin. It has been used for centuries as a spice and a medication (Shishodia et al., 2005). Apparently, this plant has anti-inflammatory, antioxidant, anticancerogenic, antiangiogenic, and cardioprotective properties (Shishodia et al., 2005; Goel et al., 2008). Research has shown that turmeric can downregulate the expression of pro-inflammatory cytokines, such as TNFα, IL-6, IL-8, IL-12, and receptors for an epithelial growth factor (EGF) and HER2/neu (Shishodia et al., 2005). Turmeric also exerts its suppressive influence on transcription factors, such as NF-κB, STAT3, PPAR γ, or beta-catenin, and on pro-inflammatory enzymes, namely, COX-2, 5-LOX, and iNOS (Shishodia et al., 2005; Hatcher et al., 2008). What is important from the point of psoriasis, turmeric induces apoptosis (Shishodia et al., 2005). There is evidence that turmeric has been used in the treatment of psoriasis and also NDs and has been investigated in such diseases in clinical trials (Shishodia et al., 2005; Hatcher et al., 2008). There are reports that turmeric may inhibit the accumulation of amyloid in AD patients and cognitive impairment (Shishodia et al., 2005). Mollazadeh et al. review the ability of turmeric to induce the production of anti-inflammatory IL-10, which takes part in the pathogenesis of AD, PD, and psoriasis (Mollazadeh et al., 2019). Another plant that was mentioned in available literature in relation to psoriasis and NDs—*Scrophularialucida*. Zengin et al. investigated the extracts from roots and aerial parts of *S. lucida*. Due to its antioxidant, anti-inflammatory, and enzyme inhibitory properties, it has been widely used in the treatment of different skin diseases, including psoriasis, but may also be useful in NDs (Zengin et al., 2019). The authors paid special attention to the ability of *S. lucida* to inhibit acetylcholinesterase and butyrylcholinesterase and inhibitors of such enzymes are used in the therapy of AD (Zengin et al., 2019). The third plant was Pongamia and Derris, of similar properties as the previous two. It was described as successful in the treatment of psoriasis and AD (Goel et al., 2021). The fourth substance was aloe-emodin, anthraquinone derived from different herbs. Aloe-emodin may exert neuroprotective influence and be useful in AD due to its anti-inflammatory, antioxidant, and acetylcholinesterase-inhibiting properties (Dong et al., 2020). It was also investigated in psoriasis due to anti-inflammatory, pro-apoptotic, and cell proliferation–inhibiting activity (Dong et al., 2020). The role of celastrol, a Chinese medical plant derivative, was described by Venkatesha et al. It is involved in the following cell signaling pathways, which are documented to be relevant in psoriasis pathogenesis and are mediators of neurodegenerative processes at the same time: NF-κB pathway, MAPK, JAK/STAT, and PI3K/Akt/mTOR (Kempuraj et al., 2016; Lin and Huang, 2016; Venkatesha and Moudgil, 2016). Moreover, it has antioxidant properties and affects cell proliferation, apoptosis, proteasome activity, angiogenesis, and immune responses (Venkatesha and Moudgil, 2016). Therefore, its preventive and therapeutic effects have been observed, among others, in psoriasis and NDs, including PD, AD, and ALS. Wei et al. assessed the efficacy of the optimized yinxieling formula (OYF), which is a Chinese medicinal formula successfully used in psoriasis therapy, in PD (Wei et al., 2018). They found beneficial OYF influence on the suppression of inflammation and immunomodulatory properties and suggested it could be useful also in PD (Wei et al., 2018). One article analyzed the immunosuppressive properties of artemisinin-type drugs in the treatment of different inflammatory and autoimmune diseases, including psoriasis and AD (Efferth and Oesch, 2021). Artemisinin is derived from *Artemisia annua* and it was mainly used to treat malaria but due to its pleiotropic properties, it has been introduced to many more diseases. It was suggested that artemisinin and its derivatives could be efficient in AD because it improved spatial memory and pathological features in the hippocampus and cortex, decreased neuritic plaque burden, and the β-secretase activity. Moreover, artemisinin lead to amyloid polypeptide disaggregation and inhibition of the release of pro-inflammatory cytokines (IL-1β, IL-6, TNF-α). As for psoriasis, the influence of artemisinin is uncertain, but in the experiment with imiquimod-induced psoriasis treated with artesunate systemic inflammation was inhibited, T lymphocytes number in draining lymph nodes, cumulative score, epidermal thickening, and proliferation rate (Ki-67 expression) decreased (Efferth and Oesch, 2021).

A receptor-interacting serine-threonine kinase 1 (RIP1) is involved in the regulation of necroptosis and inflammatory processes. Their meaning was established in the pathogenesis of NDs and psoriasis, and there have been clinical trials with RIP1 inhibitors as potential drugs started a while ago (Harris et al., 2019; Newton, 2020).

An interesting point of view was presented by Man et al. who described the term “inflammaging” which is related to chronic, low-grade inflammation in aging subjects, which leads to the development of metabolic disorders and AD (Man and Elias, 2019). These researchers pointed out psoriasis as one of the diseases which may be a factor contributing to age-related systemic conditions mentioned above. They assumed that inflammation of the skin, which is an organ of a great surface, may lead to cytokines release into the bloodstream and result in inflammaging disorders development (Man and Elias, 2019).

### The role of bacteria

It has been suggested that alterations in the gut microbiome are involved in the pathogenesis of different inflammatory conditions, including NDs and psoriasis (Polkowska-Pruszyńska et al., 2020). Physiological intestinal microflora influences the balance between Th1, Th2, and Th17 lymphocytes subpopulations. Changes in gut colonization may trigger chronic inflammatory conditions (Polkowska-Pruszyńska et al., 2020). There was also a concept of the ‘gut–brain–skin axis' proposed, which explained how mental disorders may regulate the skin condition. It seems that the gut microbiome can mediate the cross talk between the immune and nervous systems *via* the secretion of neurotransmitters in psoriasis (Chen et al., 2021b). At the same time, the role of “gut–brain–axis” has been described in the pathogenesis of some NDs, mainly PD. It appears that intestinal flora affects the maturation and function of tissue-resident immune cells in the central nervous system and also the activation of peripheral immune cells. This way it may influence the pathological process of neuroinflammation, brain injury, and neurogenesis (Blum, 2017). Based on such information, we may assume that the gut microbiome could be an important factor contributing to nervous tissue and skin function, and all these three entities may affect each other, which surely requires further research.

Bacterial biofilm, which is a cluster of cells that attach to biotic or abiotic surfaces (Mirzaei et al., 2020), could be another factor involved in NDs and psoriasis pathogenesis. Apparently, there is such biofilm in the brain of patients with AD, which is located in the early pathological plaques of brain samples in an extracellular state (Mirzaei et al., 2020). As for psoriasis, in which infection is an important environmental factor, and can trigger or exacerbate the symptoms, persistent group A *Streptococci* infections are present in psoriatic plaques but it is difficult to grow a culture and antistreptolysin is often not elevated, which could be explained by the evasion of the immune cell response through the formation of biofilms inside tonsils (Mirzaei et al., 2020). This could suggest another similar link in both disorders and indicate future research ideas.

An interesting matter is the cell wall–deficient microbes (L-forms), which are present in human blood and until they remain in balance with the host homeostasis it is perceived as eubiotic (Markova, 2020). However, when an excess of cell wall–deficient microbes occurs, there is an imbalance called dysbiosis. Researchers described the presence of dysbiotic blood microbiota in subjects with psoriasis and PD, which suggest the disease-trigger potential of cell wall–deficient present in the blood. The authors stated that live and metabolically active bacterial and fungal cell wall–deficient microbes may cause the release of microbial byproducts and toxins, which happen to have additional immunosuppressive, tissue-toxic, or allergic properties, and may as well potentially trigger autoimmune disorders (Markova, 2020).

*Mycobacterium Avium Paratuberculosis* (MAP) subspecies were also taken into account in regard to psoriasis and NDs (Ekundayo and Okoh, 2020); however, it has not been fully established. Considering the undeniable role of autoimmune mechanism in psoriasis and the association of MAP with such disorders, the possible role of MAP in the pathogenesis of this dermatosis has been suggested, but it has not been investigated in medical experiments, so its role is uncertain. More research, although still insufficient, was performed in the case of PD and MAP association, e.g., MAP DNA and antibodies against MAP were detected in PD subjects, but still, this matter remains inconclusive and requires further investigation (Ekundayo and Okoh, 2020).

### Metabolic disorders

The association between psoriasis and MS is probably one of the most thoroughly studied. Psoriasis is associated with an increased risk of obesity, arterial hypertension, diabetes mellitus, dyslipidemia, and non-alcoholic fatty liver disease (NAFLD) (Nowowiejska et al., 2020). Moreover, the efficient therapy of mentioned disorders leads to the improvement of psoriatic skin lesions (Nowowiejska et al., 2020). Noteworthy, there is also evidence for the role of MS in the pathogenesis of NDs, namely AD (Halmos and Suba, 2017). The insulin resistance in the brain promotes neurodegenerative processes, which may be additionally exacerbated by ROS (Halmos and Suba, 2017). Moreover, MS is also associated with the occurrence of obstructive sleep apnea syndrome (OSAS) (Borel, 2019), and our team has proved that psoriatics are at greater risk of OSAS than persons without this dermatosis (Nowowiejska et al., 2021). The association should also be perceived the other way round since OSAS has been determined to have unfavorable affect psoriasis, and as for NDs, such observation was made for PD (Chen et al., 2021a), so it appears their another, common comorbidity.

### The efficacy of common drugs in the treatment of psoriasis and neurodegenerative diseases

We found numerous articles describing successful treatment with the same agent in psoriasis as well as another ND. As this could implicate associations between them, we would like to analyze them more thoroughly.

In addition to the involvement of particular cytokines in the pathogenesis of psoriasis and NDs, we would also like to address the issue of drugs targeted against particular molecules, which are used in psoriasis but turned out to be also beneficial in different NDs. First, we would like to discuss the use of anti-TNFα agents, which are biologics widely administered for psoriasis as this cytokine is an important factor in psoriasis pathogenesis. We managed to find a few articles describing beneficial effects on the course of NDs in patients receiving anti-TNFα agents for psoriasis. The most valuable study was described earlier—their team found out that treatment of psoriatics with anti-TNFα agents decreases the risk of AD (Zhou et al., 2020). Bassi et al. reported a case of a patient with an unspecific mental disability who received anti-TNFα agent—etanercept—for psoriasis to which she responded rapidly and within half a year her skin lesions resolved completely. What is curious about this case, is the fact that she improved psychologically and neurologically. These authors mentioned also other cases where the administration of anti-TNFα agents was successful, among others in AD, so they postulate common pathogenesis of psoriasis and AD in terms of TNFα participation (Bassi and De Filippi, 2010). An opposite case was described by Bougea et al. who reported a patient with psoriatic arthritis treated with another anti-TNFα agent—adalimumab—and developed ALS (Bougea et al., 2014).

A positive influence of GLP-1 (gut-derived glucagon-like peptide-1) receptor agonists, which are approved for the treatment of type 2 diabetes mellitus, was suggested (Lee and Jun, 2016; Nauck et al., 2021). The idea of the beneficial use of GLP-1 receptor agonists in NDs comes from the discovery that GLP-1 receptor signaling is involved in cognitive functions and that such receptor agonists can induce neuronal growth and synaptic plasticity as well as reduce apoptosis and oxidative stress. Since the last two paths are involved in psoriasis pathogenesis, GLP-1 receptor agonists turned out to beneficially influence skin lesions severity. GLP-1 receptor agonists have been investigated in AD and PD. In clinical trials on patients with AD who were given GLP-1 receptor agonists an improvement in brain region connectivity was observed, but unfortunately not in cognitive impairment. Nevertheless, new trials are going on. As for PD, the use of GLP-1 receptor agonists for motor deficits is of low certainty, but there is evidence for the protective influence of such agents on dopaminergic neurons in mice models. In psoriatic patients the positive influence of GLP-1 receptor agonists was observed only in subjects with both, psoriasis and diabetes mellitus, but not in subjects with psoriasis without diabetes (Nauck et al., 2021).

Another example of the use of antidiabetic drugs is thiazolidinediones—peroxisome proliferator-activated receptor (PPAR) γ agonists (Menendez-Gutierrez et al., 2012). The role of these drugs in psoriasis was reviewed and suggested that they inhibit the proliferation of epidermal cells *via* inhibiting the autophosphorylation of epidermal growth factor receptor, signaling of extracellular signal-regulated kinases/mitogen-activated protein kinase (ERK/MAPK) and inhibition of transforming growth factor (TGF)-β in keratinocytes. As for PPAR β/δ, research has shown that its mRNA is elevated in psoriatic skin lesions and also expressed in T lymphocytes from psoriatic skin and blood. PPAR β/δ promotes the proliferation of T lymphocytes and inhibits their apoptosis, which results in persistent T cells infiltration in psoriatic skin; therefore, in this case, administration of PPAR β/δ inhibitors could be potentially beneficial. As for the role of PPAR in NDs, it appears that ligands of PPAR β/δ and PPAR γ have antioxidant properties and protect neuronal cells from apoptosis, as well as reduce the inflammatory activation of astrocytes and microglia and their production of pro-inflammatory cytokines and NO, which exert neurotoxic influence, but the nature of PPAR β/δ is opposite in NDs and psoriasis (Menendez-Gutierrez et al., 2012).

Phosphodiesterases 4 (PDE4) are enzymes that are engaged in cAMP (cyclic adenosine monophosphate) degradation. Therefore, their inhibitors are able to influence cAMP levels and affect leukocytes' activity (including inflammatory processes) and neurotransmitter signaling through adenylyl cyclase-linked G-protein coupled receptors (Houslay et al., 2005). Due to such properties, these agents have become useful in the treatment of psoriasis (apremilast) and may become also in AD, ALS, HD, and PD (Houslay et al., 2005; Bhat et al., 2020).

The beneficial role of dimethyl fumarate (DMF) in NDs has been shown (Rosito et al., 2020), whereas DMF is a known drug used in the systemic treatment of psoriasis. It has cytoprotective and antioxidant properties; it acts *via* activation of NRF2, NF-kB transcription factors, and the glutathione antioxidant pathway and also the regulation of the iron homeostasis of the brain. Research has shown that DMF may directly influence axonal preservation and remyelination, by modifying regulatory thiols on several proteins; therefore, it is suggested that it might be useful in PD, AD, and ALS (Rosito et al., 2020). For instance, it has been proved that therapy with DMF significantly reduced cell death caused by amyloid beta-induced accumulation, as well as tau protein phosphorylation and intracellular ROS production in AD *in vitro* models. In PD, DMF has been observed to exert a protective influence against alfa synuclein toxicity and decrease micro- and astrogliosis. As for ALS, it has been reported that outcomes of clinical trials of DMF in ALS patients seem promising (Rosito et al., 2020).

We found four reports where resolution or improvement in psoriatic skin lesions was observed after administration of levodopa (Barbeau, 1970; Barbeau and Giroux, 1972; Giroux et al., 1972; Rojo Suárez et al., 2017). Levodopa is a dopaminergic drug used as a first-line treatment option for PD (Hayes, 2019). Barbeau et al. noticed the resolution of skin lesions in patients with PD and psoriasis treated with levodopa (Barbeau and Giroux, 1972). Giroux et al. encouraged by these observations decided to perform their own original study where they administered levodopa with the peripheral DOPA decarboxylase inhibitor to psoriatic patients without PD. They observed an improvement in skin condition after 4 months (from satisfactory to excellent). They suggested that it results from levodopa being cAMP cascade-stimulating agent, which inhibits cell proliferation (Giroux et al., 1972). Rojo Suárez et al. described a case report of a patient with intertriginous psoriasis, resistant to standard therapy, and PD who was prescribed levodopa. First, they observed gradual improvement of skin and neurological condition and later relapse when the patient stopped taking her medications and again the resolution of skin lesions after reintroduction of levodopa (Rojo Suárez et al., 2017). Moreover, they discussed several similar studies. Barbeau in his review also pointed out the potential role of dopamine and levodopa treatment in other NDs, namely ALS, but with no specific results or explanations (Barbeau, 1970).

There is also a suggestion that NF-κB, a known pro-inflammatory transcription factor, may be inhibited by chondroitin sulfate, hence, this agent could become a therapeutic option in AD, PD, or psoriasis (Vallières and du Souich, 2010).

The potential use of retinoids in NDs has been postulated (Tippmann et al., 2009; Lerner et al., 2012; Lauer et al., 2021). Acitretin, one of the II-generation retinoids, is frequently used in psoriasis (Nowowiejska et al., 2020). It influences epidermal cell proliferation and differentiation which is why it has been successful in psoriasis therapy (Lerner et al., 2012). Retinoids have some other properties though, including antioxidants, involvement in neurite outgrowth, and direct inhibition of amyloid formation. Different agents have been analyzed in terms of AD treatment, particularly acitretin, all-trans retinoic acid, and synthetic retinoid AM-80. Acitretin, which is used in psoriasis as mentioned, acts due to the upregulation of metalloproteinase ADAM10, which can activate α-secretase and shift amyloid metabolism away from amyloidogenic fragments formed by cleavage of the amyloid peptide from amyloid precursor protein (Lerner et al., 2012). Lauer et al. performed a study on mice treated with acitretin and found out that this agent leads to an increase in non-amyloidogenic amyloid-precursor-protein-processing, prevents amyloid production, and elicits improvement in cognitive functions in AD mice models (Lauer et al., 2021).

**Figure 3:**
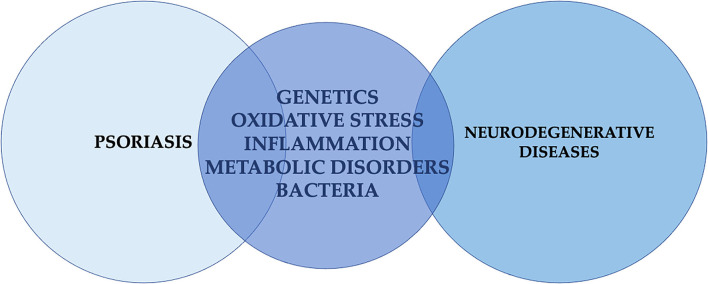
Main common factors associated with psoriasis and neurodegenerative diseases.

The main common factors associated with psoriasis and NDs are presented in Figure 3.

There are several limitations to our review. First, there is a paucity of original data on this matter; therefore, we cannot draw conclusions that could indicate the formulation of recommendations for psoriatic patients. We included many articles with even the slightest reference to a possible association between psoriasis and NDs—on one hand, it gives us a bigger picture and inspiration for future studies, on the other hand, these are not all high-quality evidence. At last, we included only three chosen NDs, but it is also possible that psoriasis is associated with other diseases from this group.

## Conclusion

Psoriasis is characterized by great comorbidity associated with different body organs and systems, so it is reasonable to analyze the possible connection of this dermatosis also with neurodegenerative diseases, which have not been widely studied so far. Probably, the majority of available scientific evidence concerns the relationship of psoriasis with Alzheimer's disease and Parkinson's disease. Based on the evidence from the original studies and reviews, which investigated the risk of neurodegenerative diseases in psoriatic patients, it seems that the risk of Parkinson's disease in psoriatics is not increased, and the evidence for increased risk of Alzheimer's disease slightly prevails the data that state the opposite, although we have to keep in mind that there are only 7 original studies specifically investigating this matter. Very little is known about the association of psoriasis with amyotrophic lateral sclerosis and original data do not support such a relationship. On the other hand, we analyzed many common pathogenetic factors involved both in psoriasis and neurodegenerative diseases and it could be possible that these entities are associated with each other but at this moment there is not too much strong evidence to support this assumption. This potentially common background includes mainly shared genetic factors, similar inflammatory cytokines and cell profiles involved, oxidative stress phenomenon, and association with metabolic disorders. At the same time, the analysis of particular drugs, which are effective in the treatment of both, neurodegenerative diseases and psoriasis, in most cases indicate rather pleiotropic activity of these agents than the influence on the same pathogenic components. Nevertheless, studies may suggest that systemic treatment for psoriasis could be studied as potential activity contributing to the reduction of Alzheimer's and Parkinson's disease risk. The paucity of original research prevents from drawing definitive conclusions and providing guidelines but it seems reasonable to perform more in-depth original research to confirm or deny these associations and perhaps form new recommendations for psoriatic patients.

## Author contributions

Conceptualization, investigation, writing—review and editing, and project administration: JN and AB. Methodology, data curation, resources, writing—original draft preparation, and visualization: JN. Validation: IF. Supervision: AB and IF. All authors have read and agreed to the published version of the manuscript.

## Conflict of interest

The authors declare that the research was conducted in the absence of any commercial or financial relationships that could be construed as a potential conflict of interest.

## Publisher's note

All claims expressed in this article are solely those of the authors and do not necessarily represent those of their affiliated organizations, or those of the publisher, the editors and the reviewers. Any product that may be evaluated in this article, or claim that may be made by its manufacturer, is not guaranteed or endorsed by the publisher.
