# Supplementary material for: Psoriasis and neurodegenerative diseases—a review
Source: Front Mol Neurosci. 2022 Sep 26;15:917751. doi: 10.3389/fnmol.2022.917751 (PMC9549431; doi:10.3389/fnmol.2022.917751)
Supplement: Supplementary file 1 [file Table_1.docx]

Supplementary Material

# Supplementary Table

**Supplementary Table 1. List of papers took into consideration and their key outcomes.**

| **No** | **Author** | **Year** | **Type of paper** | **Analyzed/ mentioned ND** | **Key observation** |
| --- | --- | --- | --- | --- | --- |
| Existing original studies directly regarding the association between psoriasis and neurodegenerative disorders | | | | | |
| 1 | Kim et al. | 2020 | Original | AD | the incidence of AD was significantly increased in patients with psoriasis compared to controls without psoriasis; treatment for psoriasis was associated with reduced risk of AD |
| 2 | Zhou et al. | 2020 | Original | AD | psoriasis associated with higher risk of AD and dementia; anti-TNFα agents were associated with decreased risk of comorbid AD in patients with psoriasis |
| 3 | Lai et al. | 2021 | Original | AD | no significant influence of psoriasis on the time of AD onset in patients with Down’s syndrome |
| 4 | Pezzolo et al. | 2021 (Epub 2018) | Original | AD | psoriasis was not associated with preclinical markers or higher risk of dementia |
| 5 | Wotton et al. | 2017 | Original | AD among others | risk of AD was elevated in patients admitted to hospital with psoriasis |
| 6 | Yokoyama et al. | 2016 | Original | AD | genetic overlap between AD and immune-mediated diseases suggest that immune system processes influence AD pathogenesis and progression; similar pattern of enrichment for PSOR SNPs conditional on AD SNPs, suggesting symmetric genetic enrichment between AD and the immune-mediated diseases |
| 7 | Li et al. | 2021 | Original | ALS, PD, AD | no shared genetic loci found between psoriasis and ALS, AD, PD in GWAS |
| 8 | Witoelar et al. | 2017 | Original | PD | PD may be associated with autoimmune diseases but genetic enrichment between PD and psoriasis was found to be weak and decreased risk of psoriasis (although statistically insignificant) |
| 9 | Bartkiewicz et al. | 2017 | Original | PD | no statistically significant differences in prevalence of NDs in general between patients with bullous pemphigoid and psoriasis |
| 10 | Lee et al. | 2020  (Epub 2019) | Original | PD | psoriatics showed significantly increased risk of PD. The risk of PD was significantly higher among the psoriasis patients not receiving systemic therapy and lower among the psoriasis patients on systemic therapy |
| 11 | Sheu et al. | 2013 | Original | PD | psoriatics were found to be at significant risk of parkinsonism during a 5-year follow-up |
| Existing review papers regarding the association between psoriasis and each neurodegenerative disorder separately or neurodegenerative disorders in general | | | | | |
| 12 | Zhao et al. | 2021 | Review | AD | most studies supported the hypothesis that psoriasis is a risk factor for dementia. However, well-designed stratified cohort studies assessing both psoriasis severity and treatment status are still required |
| 13 | Zhang et al. | 2021 | Review | AD | psoriasis and AD share the same factors in pathogenesis: genetic and inflammatory |
| 14 | Amanat et al. | 2018 | Review | PD | inconsistent reports regarding increased risk of PD in psoriatics |
| 15 | Ungprasert et al. | 2016 | Review | PD | statistically significant increased risk of PD among patients with psoriasis |
| Genetics | | | | | |
| 16 | Zhang et al. | 2011 | Review | AD, PD | the role of ApoE in modulation of inflammation and oxidation both in psoriasis and NDs |
| 17 | Zhou et al. | 2021 | Original | NDs in general | mutations in Zdhhc family genes alter palmitoylation or de-palmitoylation which may result in ND and psoriasis |
| 18 | Woo et al. | 2010 | Original | AD | chromosome territory reorganization may play a role in common human diseases such as AD and psoriasis |
| 19 | D'Amico et al. | 2017 | Original | ALS | association between the rs2294020 SNP and susceptibility to psoriasis but not ALS |
| 20 | Theotoki et al. | 2020 | Review | PD | dicer upregulation in PSO and downregulation in PD |
| Oxidative stress | | | | | |
| 21 | Srivastava et al. | 2017 | Review | AD, PD | oxidative stress as a cause of NDs and psoriasis |
| 22 | Maes et al. | 2011 | Review | AD, PD | microglial activation has mutual influences with AD, PD and with peripheral cell-mediated immune activation and/or inflammation, and oxidative and nitrosative stress, which may be induced, among others, by psoriasis |
| 23 | Chiurchiù et al. | 2011 | Review | AD, PD, ALS | both skin and central nervous system are sensitive to oxidative stress; important role of oxidative stress involved in pathogenesis of AD, PD, ALS and psoriasis |
| 24 | Gerbaud et al. | 2005 (Epub 2004) | Review | AD | increase in manganese superoxide dismutase and Cu,Zn-SOD functional activity in human dermal psoriatic fibroblasts and involved in AD pathogenesis |
| 25 | Li et al. | 2011 | Review | ND in general, AD | the level of manganese superoxide dismutase, key enzyme protecting the energy-generating mitochondria from oxidative damage, is reduced in NDs and psoriasis |
| 26 | Jaganjac et al. | 2020 (Epub 2019) | Review | AD, PD | induction of intracerebral oxidative stress and lipid peroxidation in ND and psoriasis |
| 27 | Nurminen et al. | 2011 | Original | AD | vascular adhesion protein-1 (VAP-1) is linked to AD and psoriasis, and selective inhibitors of VAP-1 could potentially be used to therapy of those diseases |
| 28 | Das | 2005 | Review | AD | low concentration of PUFA in patients with psoriasis and AD which leads to activation of ACE and high content of angiotensin II which stimulates the release of pro-inflammatory cytokines, activates NF-κB, increases oxidative stress |
| 29 | Das | 2005 | Review | AD | angiotensin-II may play a significant role in AD and psoriasis |
| Inflammation | | | | | |
| 30 | Schultze et al. | 2018 | Review | NDs in general | the same chronic inflammatory background in ND and psoriasis |
| 31 | Chen et al. | 2020 | Review | AD, PD, ALS | important role of IL-17 both in pathogenesis of psoriasis and ND |
| 32 | Bougea et al. | 2014 | Letter to editor describing case report | ALS | ALS developing during adalimumab therapy for psoriatic arthritis (PsA); the spinal cord of ALS patients shows a milieu in which polarization of CD3 cells to IL-17A-producing cells can develop in response to products of ages, T-cells; including IL-1b, TNF-a. Activation of chronic inflammation, including the IL-17A mediated pathway, occurs also in PsA |
| 33 | Mollazadeh et al. | 2019 | Review | AD, PD | important role of IL-10 both in pathogenesis of psoriasis and ND basing on immune modulation by curcumin |
| 34 | Xin et al. | 2015 | Review | AD | involvement of IL-22 in AD and psoriasis (mentioned study Saresella et al. [52]) |
| 35 | Yu et al. | 2008 | Original | AD | TNFα-mediated inflammatory pathways involved in psoriasis and AD |
| 36 | Harris et al. | 2019 | Original | AD, ALS | RIP1 kinase, which regulates necroptosis and inflammation, may play an important role in psoriasis and AD pathogenesis and RIP1 kinase inhibitors are tested in therapy of these diseases |
| 37 | Newton et al. | 2019 | Review | AD, ALS | inhibition of receptor-interacting serine threonine kinase 1 (RIPK1) pharmacologically or genetically is reported as beneficial in AD, ALS and psoriasis |
| 38 | Tajti et al. | 2020 | Review | AD, PD | potential beneficial role of venom peptides targeting K_V_1.3, in particular from sea anemones and scorpions in psoriasis and NDs |
| 39 | Bencherif et al. | 2011 | Review | PD | increased levels of cytokines such as TNF-α, IL-1 β, and interferon-γ are present in the substantia nigra of PD patients, along with proof of important role played by α7 receptors in PD pathogenesis; at the same time perivascular expression of the α7 nicotinic receptor was detected in the majority of psoriatics with arthritis, underscoring a potential role for α7 receptors and the cholinergic inflammatory pathway in proliferation of endothelial cells in the inflamed synovium of these patients |
| 40 | Robertson et al. | 2018 | Letter to editor | AD | role of C5a in NDs such as AD and psoriasis; C5a could be a new therapeutic approach |
| 41 | Storelli et al. | 2019 | Review | PD | peripheral T lymphocytes, which are proinflammatory CD4+ T cells, seem to be key players in PD and psoriasis |
| 42 | Schultze et al. | 2015 | Review | NDs in general | macrophages play an important role in diseases associated with chronic inflammation, e.g. psoriasis and ND |
| 43 | Shishodia et al. | 2013 | Review | NDs in general | influence of turmeric on genes and its possible role both in NDs and psoriasis |
| 44 | Shishodia et al. | 2005 | Review | NDs in general | possible beneficial role of turmeric both in treatment of NDs and psoriasis |
| 45 | Hatcher et al. | 2008 | Review | AD | possible beneficial role of turmeric both in treatment of AD and psoriasis |
| 46 | Goel et al. | 2008  (Epub 2007) | Review | AD, PD | possible beneficial role of turmeric in treatment of AD, PD and psoriasis |
| 47 | Pari et al. | 2008 | Review | AD | possible beneficial role of turmeric both in treatment of neurodegenerative diseases and psoriasis |
| 48 | Akaberi et al. | 2021 | Review | AD, ALS | beneficial role of turmeric in treatment of AD, ALS and psoriasias |
| 49 | Aggarwal et al. | 2004 | Review | AD | activation of NF-κB involved in AD and psoriasis, attempt to interrupt this pathway by phytochemicals |
| 50 | Zengin et al. | 2019 | Original | AD | *Scrophularia lucida* as a valuable antioxidant, anti-inflammatory, enzyme inhibitory properties agent in AD and psoriasis |
| 51 | Goel et al. | 2021 | Review | AD | some species of Pongamia and Derris have been found as a source of 36 flavonols and their glucosides, of which compound 39 has been investigated in treatment of AD and psoriasis |
| 52 | Dong et al. | 2020 (Epub 2019) | Review | AD | aloe-emodin – natural anthraquinone derivative and an active ingredient of Chinese herb- may beneficial in therapy of ND and psoriasis |
| 53 | Venkatesha et al. | 2016 | Review | AD, PD, ALS | beneficial role of celastrol, a triterpenoid derived from traditional Chinese medicinal plants, which has anti-inflammatory, anti-oxidant, and anti-cancer activities in therapy of psoriasis and NDs |
| 54 | Wei et al. | 2018 | Original | PD | Optimized Yinxieling Formula (OYF), a Chinese medicinal formula is used in therapy of psoriasis and could be beneficial also in PD |
| 55 | Efferth et al. | 2021 | Review | AD | artemisinin-type drugs may be beneficial in therapy of AD and psoriasis |
| 56 | Man et al. | 2019 | Review | AD | epidermal dysfunction, as in psoriasis, leads to ‘inflammaging’ as in aging individuals and skin could be a major contributor to inflammaging which predispose to AD |
| The role of bacteria | | | | | |
| 57 | Polkowska-Pruszyńska et al. | 2019 | Review | ND in general | gut microbiome alterations are involved both in pathogenesis of psoriasis and ND |
| 58 | Mirzaei et al. | 2020 | Review | AD | the role of bacterial biofilm in AD and psoriasis; studies have confirmed the presence of bacterial biofilm in the  brain of patients with AD. This biofilm has been located in the early pathological plaques of brain samples |
| 59 | Markova et al. | 2020 | Original | PD | appearance of "dysbiotic" blood microbiota that outlined the disease-trigger potential of opportunistic bacteria and fungi existing in blood as CWD variants in PD and psoriasis |
| 60 | Ekundayo et al. | 2020 | Review | PD | possible role of MAP in psoriasis and PD has been postulated; experimental/ clinical demonstrations are needed to provide evidence-based roles of MAP |
| Metabolic disorders | | | | | |
| 61 | Halmos et al. | 2017 | Review | NDs in general | association of metabolic syndrome with NDs |
| 62 | Chen et al. | 2021 | Review | PD | both psoriasis and PD may be associated with OSAS |
| The efficacy of common drugs in treatment of psoriasis and neurodegenerative diseases | | | | | |
| 63 | Bassi et al. | 2010 | Case report | AD | etanercept (anti-TNF α agent) used in therapy of psoriasis may beneficially influence AD patients’ condition |
| 64 | Nauck et al. | 2021 (Epub 2020) | Review | AD, PD | GLP-1 receptors agonists as a treatment option both in psoriasis and ND may be beneficial; GLP-1 receptor signaling is involved in cognitive functions and GLP-1 receptors agonists can induce neuronal growth and synaptic plasticity, reduce apoptosis and oxidative stress; the last two paths are involved in psoriasis pathogenesis, GLP RA turned out to beneficially influence skin lesions severity |
| 65 | Lee et al. | 2016 | Review | AD, PD | associations between psoriasis, NDs and diabetes mellitus t.2. GLP-1-based therapies show anti-inflammatory effects in ND and psoriasis |
| 66 | Menendez-Gutierrez et al. | 2012 | Review | NDs in general | administration of thiazolidinediones alleviates the symptoms of psoriasis and PPARs have positive effects  on neuronal survival of the brain and also alleviate neuroinflammation |
| 67 | Houslay et al. | 2005 | Review | AD, PD | use of PDE4 inhibitors in treatment of psoriasis, AD, mild cognitive impairment and PD |
| 68 | Bhat et al. | 2020 | Review | AD, PD, ALS | beneficial role of PDE4 inhibitors in treatment of psoriasis and ND |
| 69 | Rosito et al. | 2020 | Review | AD, PD, ALS | dimethyl fumarate and other derivatives of fumaric acid ester compounds have been used in the treatment of psoriasis and relapsing forms of multiple sclerosis (MS); DMF has been shown to exert a neuroprotective effect on the central nervous system |
| 70 | Barbeau | 1970 | Review | AD, ALS | dopamine metabolism impairment present in some NDs and beneficial effect of dopaminergic drugs on these diseases as well as on psoriasis |
| 71 | Barbeau et al. | 1972 | Letter to editor | PD | improvement of psoriatic lesions after administration of levodopa to patients with PD |
| 72 | Giroux et al. | 1972 | Letter about the original study | - | treatment of psoriatics with levodopa+Ro 4-4602 lead to improvement in half of patients |
| 73 | Rojo Suárez et al. | 2017 (Epub 2016) | Case report | - | case describing resolution of psoriatic lesions after levodopa |
| 74 | Vallières et al. | 2010 | Review | AD, PD, ALS | chondroitin sulfate might be a useful therapeutic agent in AD, PD, ALS and psoriasis |
| 75 | Lerner et al. | 2012 | Review | AD | retinoids may be useful not only in psoriasis but also in AD |
| 76 | Tippmann et al. | 2009 | Original | AD | acitretin use may be beneficial not only in therapy of psoriasis but also AD |
| 77 | Lauer et al. | 2021 | Original | AD | acitretin used in treatment for psoriasis increases non-amyloidogenic Amyloid-Precursor-Protein-(APP)-processing, prevents Aβ-production and elicits cognitive improvement in AD mouse models |
